# Niagara and Orleans County Veterinary Medical Society

**Published:** 1901-05

**Authors:** Anderson Crowforth

**Affiliations:** Secretary


Secretary.

### NIAGARA AND ORLEANS COUNTY VETERINARY MEDICAL SOCIETY.

A REGULAR meeting was held at the office of the President, Dr. W. E. Stocking, Medina, N. Y., March 23d. The minutes of the last meeting were read and approved. The Secretary reported a balance on the right side of the ledger. The question of veterinary legislation was taken up, and an animated discussion followed, in which all expressed their views. It was the general opinion that the profession had made rapid strides in advancement since the regulations for higher education came in force, and hoped the same would be maintained as the only safeguard to public health from the veterinary stand-point.

C. Mattison, of Niagara Falls, was reported by the prosecuting committee as having had a penalty of $100 and costs recovered against him for practising without being registered.

Dr. M. D. Williams read a paper on "Milk Fever," and Dr. John Liddle presented a specimen of rupture of the true stomach in a cow. The cow lived eight weeks after the injury to the organ.

These cases drew forth a lively discussion, which was enjoyed by all. This was the largest and most interesting meeting in the history of the Society. Certificates of membership were issued at this meeting. The next meeting will be in August, at Lockport.

ANDERSON CROWFORTH,
Secretary.

